# Supplementary material for: When women eat last: Discrimination at home and women’s mental health
Source: PLoS One. 2021 Mar 2;16(3):e0247065. doi: 10.1371/journal.pone.0247065 (PMC7924788; doi:10.1371/journal.pone.0247065)
Supplement: S1 Table — (PDF) [file pone.0247065.s001.pdf]

**S1 Table. Complete SRQ questionnaire (those used in SARI are marked with an asterisk)**

1. Do you often have headaches?
2. Is your appetite poor?*
3. Do you have trouble sleeping?*
4. Are you easily frightened?
5. Do your hands shake?
6. Do you feel nervous, tense, or worried?
7. Is your digestion poor?
8. Do you have trouble thinking clearly?*
9. Do you feel unhappy?
10. Do you cry more than usual?
11. Do you find it difficult to enjoy your daily activities?
12. Do you find it difficult to make decisions?*
13. Is your daily work suffering?
14. Are you unable to play a useful part in life?
15. Have you lost interest in things?
16. Do you feel that you are a worthless person?
17. Has the thought of ending your life been on your mind?*
18. Do you feel tired all the time?*
19. Do you have uncomfortable feelings in your stomach?
20. Are you easily tired?
