# Supplementary material for: When women eat last: Discrimination at home and women’s mental health
Source: PLoS One. 2021 Mar 2;16(3):e0247065. doi: 10.1371/journal.pone.0247065 (PMC7924788; doi:10.1371/journal.pone.0247065)
Supplement: S2 Table — (PDF) [file pone.0247065.s002.pdf]

S2 Table. Association between eating last and mental health in SARI, by caste group

	SRQ total score urban & rural				
	Dalit	OBC	General Caste	Brahmin	Adivasi
	(1)	(2)	(3)	(4)	(5)
Women eat last	1.359	2.106*	1.964	1.185	3.013
	(0.595)	(0.779)	(1.062)	(0.970)	(2.781)
Age categories (reference category: 25-34)					
35-44	2.561+	0.919	1.802	5.130	1.556
	(1.447)	(0.313)	(0.876)	(5.194)	(2.108)
45-65	0.822	0.805	2.557+	2.423	0.900
	(0.372)	(0.277)	(1.239)	(2.304)	(1.091)
Education categories (reference category: 0 years)					
1-8 years	1.292	0.421*	1.105	0.620	4.260
	(0.903)	(0.174)	(0.512)	(0.661)	(5.336)
9-12 years	0.807	0.586	1.350	1.623	0.609
	(0.574)	(0.241)	(0.691)	(1.766)	(0.731)
more than 12 years	2.592	0.457	1.324	2.128	7.880+
	(2.952)	(0.232)	(0.786)	(2.422)	(8.951)
Muslim	0.113	1.170	1.407	0.417	1
	(0.245)	(0.321)	(0.644)	(0.521)	(.)
Number of assets (reference category: 0 assets)					
1	1.204	1.158	0.455+	0.125	0.169
	(0.651)	(0.518)	(0.203)	(0.207)	(0.433)
2	1.264	1.245	0.329**	0.0976	0.0540*
	(0.641)	(0.684)	(0.138)	(0.148)	(0.0650)
3	2.114	1.283	0.155**	0.0966	0.951
	(1.565)	(0.649)	(0.0923)	(0.152)	(1.389)
4	0.158+	1.099	0.232*	0.401	0.124
	(0.174)	(0.690)	(0.151)	(0.638)	(0.169)
5	0.226	1.600	0.0969***	0.0212*	0.0416**

	(0.232)	(0.976)	(0.0510)	(0.0323)	(0.0460)
State (reference group: Bihar)					
Jharkhand	0.564	1.196	0.811	1.440	4.337
	(0.244)	(0.384)	(0.324)	(0.909)	(5.966)
Maharashtra	0.538	0.813	0.529*	0.823	2.379
	(0.282)	(0.372)	(0.169)	(0.857)	(2.897)
n	178	457	334	76	58

Note: Standard errors in parentheses. + p<0.1 * p<0.05 ** p<0.01 *** p<0.001. All models restricted to married women, aged 25 or over, who were assigned to answer SRQ questions. Data were collected in the states of Bihar, Jharkhand, and Maharashtra.
