# Supplementary material for: When women eat last: Discrimination at home and women’s mental health
Source: PLoS One. 2021 Mar 2;16(3):e0247065. doi: 10.1371/journal.pone.0247065 (PMC7924788; doi:10.1371/journal.pone.0247065)
Supplement: S3 Table — (PDF) [file pone.0247065.s003.pdf]

S3 Table. Women who report having to ask for permission to go a neighbor's house are less likely to eat last in their households.

	Women eat last	
	(1)	(2)
Ask for permission to go to neighbor's house	0.121***	0.118***
	(0.0283)	(0.0273)
Age categories (reference category: 25-34)		
35-44		-0.0186
		(0.0332)
45-65		-0.0274
		(0.0327)
Education categories (reference category: 0 years)		
1-8 years		-0.0464
		(0.0331)
9-12 years		-0.159***
		(0.0451)
more than 12 years		-0.169**
		(0.0611)
Muslim		-0.129**
		(0.0452)
Number of assets (reference category: 0 assets)		
1		0.0372
		(0.0504)
2		0.0490
		(0.0501)
3		0.0630
		(0.0511)
4		0.0924+
		(0.0523)
5		0.0240
		(0.0561)
Caste group (reference group: Dalit)		
OBC		0.0452
		(0.0371)
General		-0.0660+
		(0.0394)
Brahmin		-0.0910
		(0.0719)
Adivasi		-0.00419
		(0.0650)
State (reference group: Bihar)		
Jharkhand		-0.213***
		(0.0617)

Maharashtra		-0.374***
		(0.0326)
n	1216	1176

Note: Standard errors in parentheses. + p<0.1 * p<0.05 ** p<0.01 *** p<0.001. All models restricted to married women, aged 25 or over, who were assigned to answer SRQ questions. Data were collected in the states of Bihar, Jharkhand, and Maharashtra.
